# Resveratrol, an Inhibitor Binding to VEGF, Restores the Pathology of Abnormal Angiogenesis in Retinopathy of Prematurity (ROP) in Mice: Application by Intravitreal and Topical Instillation

**DOI:** 10.3390/ijms23126455

**Published:** 2022-06-09

**Authors:** Wei-Hui Hu, Xiao-Yong Zhang, Ka-Wing Leung, Ran Duan, Ting-Xia (Tina) Dong, Qi-Wei Qin, Karl Wah-Keung Tsim

**Affiliations:** 1 Joint Laboratory of Guangdong Province and Hong Kong Region on Marine Bioresource Conservation and Exploitation, College of Marine Sciences, South China Agricultural University, Guangzhou 510642, China; whuaf@connect.ust.hk (W.-H.H.); zhangxiaoyong@scau.edu.cn (X.-Y.Z.); botina@ust.hk (T.-X.D.); qinqw@scau.edu.cn (Q.-W.Q.); 2 Shenzhen Key Laboratory of Edible and Medicinal Bioresources, The Hong Kong University of Science and Technology, Hi-Tech Park, Nanshan, Shenzhen 518063, China; lkwing@ust.hk (K.-W.L.); duanran@ust.hk (R.D.); 3 Division of Life Science and State Key Laboratory of Molecular Neuroscience, The Hong Kong University of Science and Technology, Hong Kong, China

**Keywords:** resveratrol, retinopathy of prematurity, age-related macular degeneration, anti-VEGF, natural product

## Abstract

Retinopathy of prematurity (ROP) is a severe eye disease leading to blindness. Abnormal vessel formation is the pathological hallmark of neovascular ROP. In forming vessels, vascular endothelial growth factor (VEGF) is an important stimulator. The current anti-ROP therapy has focused on bevacizumab, a monoclonal antibody against VEGF, and pazopanib, a tyrosine kinase inhibitor on the VEGF receptor (VEGFR). Several lines of evidence have proposed that natural compounds may be more effective and safer for anti-VEGF function. Resveratrol, a common natural compound, binds to VEGF and blocks its interaction with VEGFR, thereafter suppressing angiogenesis. Here, we evaluate the efficacy of intravitreal injection, or topical instillation (eye drops), of resveratrol into the eyes of mice suffering from oxygen-induced retinopathy, i.e., developing ROP. The treatment of resveratrol significantly relieved the degree of vascular distortion, permeability and hyperplasia; the efficacy could be revealed by both methods of resveratrol application. In parallel, the treatments of resveratrol inhibited the retinal expressions of VEGF, VEGFR and CD31. Moreover, the applied resveratrol significantly relieved the damage caused by oxygen radicals through upregulating the level of superoxide dismutase (SOD) and downregulating the level of malondialdehyde (MDA) in the retina. Taken together, the potential therapeutic benefit of resveratrol in pro-angiogenic diseases, including retinopathy, can be considered.

## 1. Introduction

Angiogenesis is the process of new blood vessels deriving from an existing vascular network, playing a major role in the progression and development of various diseases, including tumours and inflammation. Therefore, it is under precise control [1,2]. Pathologic angiogenesis occurs in retinopathy of prematurity (ROP) or diabetic retinopathy, and it is a key factor leading to blindness [3,4]. In a healthy retina, the growth of blood vessels should be under strict control [5,6,7]; however, under abnormal conditions, e.g., hypoxia, infection or chemical burns, angiogenesis can be induced [8,9,10]. The formation of new blood vessel(s) in the retina is named as neovascularization. Abnormal neovascularization leads to severe vision impairment, and this is a key common pathway to all impairments. Functionally, angiogenesis plays a role in supplying blood flow to various cells during eye development. Thus, this should have great benefits in terms of understanding the pathophysiology of angiogenesis and identifying possible targets of angiogenic regulation in eye diseases.

The proliferation and migration of vascular endothelial cells is essential in creating new blood vessels. Vascular endothelial growth factor (VEGF) is a key factor contributing to endothelial cell proliferation and angiogenesis [11,12]. The essential role of VEGF in angiogenesis and embryonic vasculogenesis has been supported by the inactivation of a single VEGF allele in mice leading to embryonic death [1]. Thus, a major direction of present research in anti-angiogenic therapy is focusing on VEGF. VEGF exerts its effects by binding to transmembrane endothelial cell receptors with several high-affinity receptors, especially VEGF receptor 1 (VEGFR-1) (Flt-1) and VEGF receptor 2 (VEGFR-2) (KDR/Flk-1). The binding of VEGF with its receptor leads to phosphorylation of intracellular receptors, in turn inducing the relevant downstream signalling [13]. VEGF has been shown to be closely related to retinal neovascularization in animal models and in humans [14,15]. The amounts of VEGF and VEGFR in diseased retina are higher than that of normal or avascular abnormal retina. In parallel, anti-VEGF therapy, e.g., Avastin (bevacizumab), has been very successful in treating colon cancer [16], which could regulate the neovascularization [7,17].

Retinopathy of prematurity (ROP), also known as retrolental fibroplasia, is a vasoproliferative disorder of the retina and a major cause of blindness. The pathologies are triggered by limiting retinal oxygenation: the hypoxia induces overexpression of growth factors, including VEGF, and thereafter abnormal blood vessel growth [18]. Both preventive and therapeutic medications have been proposed in fighting against ROP, e.g., vitamin E [19], supplemental essential polyunsaturated fatty acids [20], angiopoietin-1 [21] and erythropoietin [22]. Nevertheless, the understanding of pathologies of therapeutic agents is limited [23]. The clinical application of VEGF-neutralizing antibody has been a promising approach against ROP [18,23,24]. However, the antibody application demonstrated various issues, e.g., high cost, frequent and invasive injection, and side effects in ocular and systemic tissues [25,26]. Another treatment of ROP is via laser, which has been considered the most promising standard as it can attenuate excessive VEGF secretion and regression of new blood vessels by ablating the retinal vessels. However, laser therapy may trigger severe complications, such as myopic shift and defects in the peripheral visual field, as well as failures in severe ROP, including insufficient pupil dilatation and poor visualization of the retina [27,28]. Thus, the development of a variety of natural compounds in reducing retinal angiogenesis is needed. 

Polydatin [29] and resveratrol [30] have been identified for binding to VEGF from a Chinese medicinal herb, Polygoni Cuspidati Rhizoma et Radix [31]. The binding interactions of resveratrol and/or polydatin to VEGF attenuated VEGF-mediated angiogenic activities, i.e., decreased the amount of VEGF able to interact with its receptors. Under this notion, we explored the potential application of resveratrol for retinal neovascularization in an in vivo angiogenetic mice model of retinal development. Moreover, the role of resveratrol in relieving the damage caused by oxygen radicals was also evaluated.

## 2. Results

### 2.1. Resveratrol Regulates Retinal Vascular Permeability

**Figure 1:**
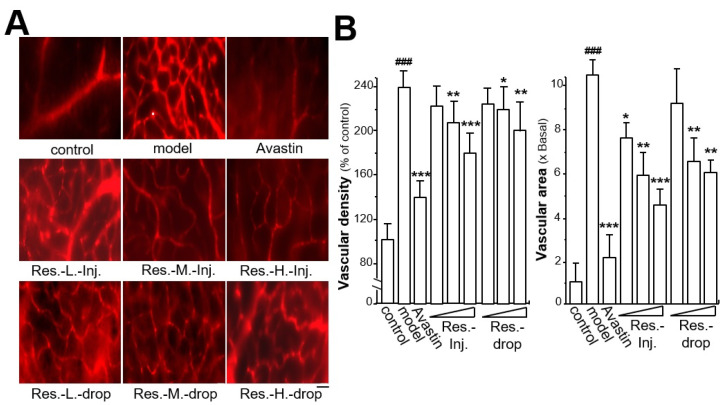
**Resveratrol reduces vascular permeability in retinal ROP model.** The groupings were: the control group, model group, Avastin group (20 mg/kg), resveratrol low-dosage group (intravitreal injection, 5 mg/kg; Res.-L.-Inj.), resveratrol middle-dosage group (intravitreal injection, 25 mg/kg; Res.-M.-Inj.), resveratrol high-dose group (intravitreal injection, 50 mg/kg; Res.-H.-Inj.), resveratrol low-dosage group (eye drop, 5 mg/kg; Res.-L.-drop), resveratrol middle-dosage group (eye drop, 25 mg/kg/day; Res.-M.-drop), resveratrol high-dose group (eye drop, 50 mg/kg/day; Res.-H.-drop). (**A**) Red fluorescence represents the blood vessels. (**B**) Image-Pro Plus 6.0 software was used for quantification of retinal vascular density (left) and area (right). The data are expressed of the percentage of control or the fold of change of control (x basal), in mean ± SEM, where *n* = 6; ### *p* < 0.001 vs. the control group; * *p* < 0.05; ** *p* < 0.01; *** *p* < 0.001 vs. the model group. Bar = 25 μm.

To investigate the role of resveratrol in retinal vascular permeability in mice, an ROP mice model was developed. The efficacy of resveratrol on vascular permeability was determined separately by intravitreal injection or topical instillation. Avastin was selected as a positive control as it is known to inhibit vascular permeability [32]. Neonatal mice were exposed to oxygen (75 ± 2%) in a hyperoxic chamber and then returned to normal air. In control mice, vascular morphology was unchanged, and no blood leakage was observed. In ROP mice, obvious vascular distortion was revealed, and hyperoxia induced vascular permeability by increasing vascular density and area (Figure 1A,B). Compared with the control group, the vascular density was increased by ~140% in the hyperoxia-treated model group, and the vascular area was enlarged by over 10-fold. These robust changes demonstrated the establishment of the retinal ROP mouse model. The vascular permeability was restored in drug-treated mice by application of Avastin and resveratrol. Applied Avastin markedly decreased the hyperoxia-induced vascular density by ~100%, serving as a positive control (Figure 1A,B). The treatment of resveratrol attenuated vascular permeability in a dose-dependent manner. As shown in Figure 1A,B, resveratrol application by intravitreal injection at a high dose demonstrated the maximal suppressive effects on vascular density, down from ~240% to ~175%; resveratrol application by eye drops at a high dose demonstrated maximal inhibitory effects on vascular density down to ~200%. The vascular density of mice treated with resveratrol by intravitreal injection was lower than that in groups treated with resveratrol by topical instillation. The suppressive effects of vascular density in groups that were resveratrol-treated by intravitreal injection at low and middle doses were 40% and 65%, respectively. In groups that were resveratrol-treated by topical instillation at low and middle doses these values were 20% and 40%, respectively (Figure 1A,B). 

Drug treatment exerted inhibitory effects on vascular area enlarged by hyperoxia (Figure 1A,B). Avastin significantly reduced the enlarged vascular area by ~10-fold. The suppressive rates of resveratrol on vascular area were significantly increased, dose-dependently, in comparison with the model group (Figure 1A,B). The treatment of resveratrol by intravitreal injection at a high dose demonstrated the highest rate of inhibition (from ~10-fold to ~4-fold) among the drug-treated groups, and resveratrol treatment at low and middle doses via same method of administration decreased vascular area by ~3-fold and ~5-fold, respectively (Figure 1A,B). The inhibitory rates of vascular area in the groups that were resveratrol-treated by eye drops at middle and high doses were ~2-fold to ~6-fold, respectively. The application of resveratrol by eye drops at a low dose inhibited vascular density and area slightly, but not at a significant standard.

### 2.2. Resveratrol Relieves Vascular Hyperplasia in ROP Mice

**Figure 2:**
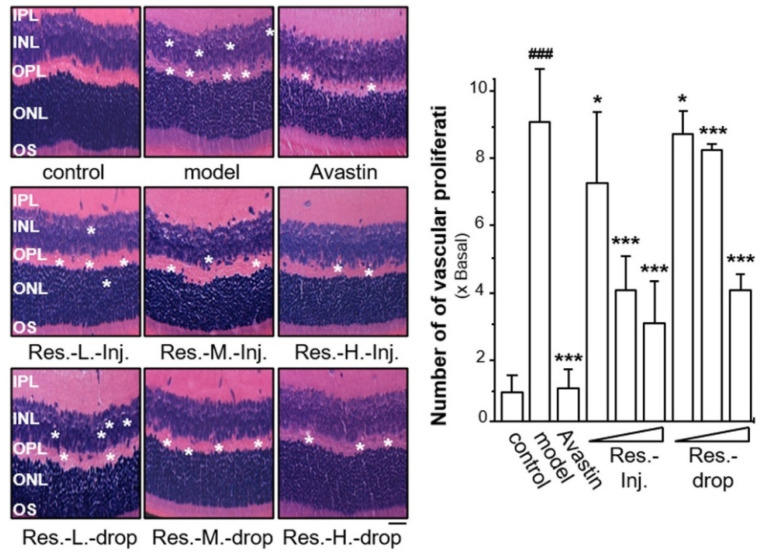
**Resveratrol decreases the degree of vascular hyperplasia in retinal ROP model.** Retinal tissues from drug-treated mice were collected, as in Figure 1. Tissues were processed with hematoxylin and eosin (H&E) staining. Blue or purple blue colour represents the nucleus, pink colour represents the cytoplasm, and bright red colour represents the red blood cells (left). * shows the part of vascular hyperplasia. ImageJ software was used for the quantification of microvessel density (right). Zen and Image J software were used to quantify images of vascular hyperplasia regions (right panel). IPL (inner plexiform layer), INL (inner nuclear layer), OPL (outer plexiform layer), ONL (outer nuclear layer) and OS (outer segment) are shown. The results are expressed as the fold of change to the control (x basal), where the control group (no drug) was set as 1, Mean ± SEM, where *n* = 6; ### *p* < 0.001 vs. the control group; * *p* < 0.05; *** *p* < 0.001 vs. the model group. Bar = 25 μm.

During ROP model establishment, retinal vessels are developed due to being exposed to hyperoxia. Upon returning to normal room air, relative hypoxia contributes to abnormal neovascular tufts in the retina. These tufts abnormally protrude from the surface of retina into the retinal vitreous, causing vascular hyperplasia [33]. Here, the role of resveratrol in formation of vascular hyperplasia in the ROP model was determined by retinal H&E staining. The hyperoxia induced the degree of vascular hyperplasia up to ~9-fold in model mice (Figure 2). The application of resveratrol at various doses, given by intravitreal injection and eye drops, significantly suppressed vascular hyperplasia in dose-dependent manners. The maximal suppression was demonstrated after a high dose of resveratrol given by intravitreal injection, reducing the vascular hyperplasia down to ~3-fold that of the control (Figure 2). The application of resveratrol by intravitreal injection at middle and low doses reduced vascular hyperplasia to ~7-fold and ~4-fold, respectively. Resveratrol application at a high dose, given by eye drops, exerted a decrease in vascular hyperplasia by ~4-fold that of the control, while the low and middle doses decreased vascular hyperplasia to ~8.5-fold and ~8-fold (Figure 2). The drug administration via intravitreal injection demonstrated better effects than that by topical instillation, which could be due to better penetration into the retina by direct injection. Avastin, serving as a positive control, was shown to reduce vascular hyperplasia to control levels.

### 2.3. Resveratrol Inhibits Expression of VEGF, VEGFR2 and CD31

**Figure 3:**
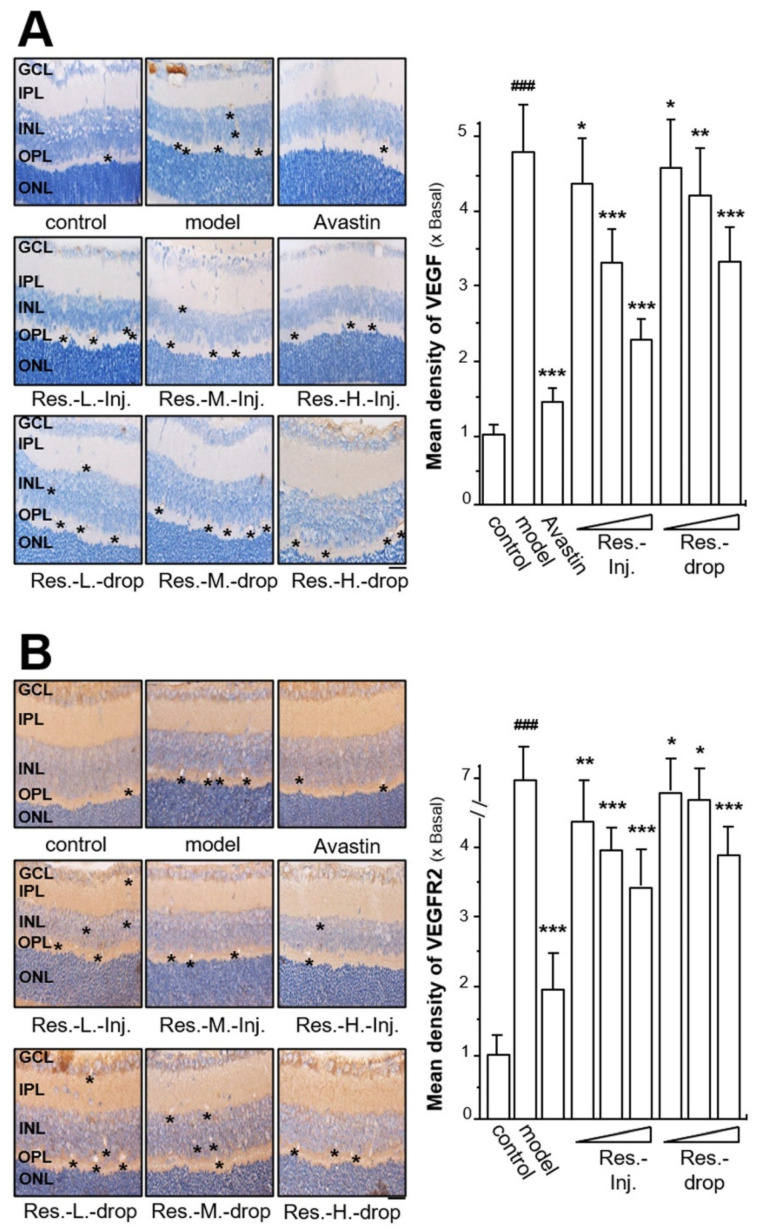
**Resveratrol inhibits hyperoxia-induced expressions of VEGF and VEGFR2 in retinal ROP model.** (**A**) Retinal tissues from drug-treated mice were collected, as in Figure 1. Tissues were immuno-stained with the VEGF antibody and stained by DAB dye. The positive area is brown or yellowish brown (left). * shows the expressed part of the target protein. Image-Pro Plus version 6.0 software was used for the quantification of VEGF density (right). (**B**) Retinal tissues were immuno-stained with the VEGFR2 antibody and stained by DAB dye. The positive area is brown or yellowish brown (left). * shows the expressed part of the target protein. Image-Pro Plus version 6.0 software was used for the quantification of VEGFR2 density (right). Results are expressed as the fold of change to the control (x basal), where the control group (no drug) was set as 1, Mean ± SEM, where *n* = 6; ### *p* < 0.001 vs. the control group; * *p* < 0.05; ** *p* < 0.01; *** *p* < 0.001 vs. the model group. Bar = 25 μm.

To determine whether resveratrol treatment affects angiogenic biomarkers in ROP pathogenesis, the expression of VEGF and VEGFR-2 in the retinal tissues was analysed by immunohistochemistry. The hyperoxia in the mouse model induced the expression of VEGF by increasing its density in retinal tissues; the induction of VEGF expression density was up to ~5-fold (Figure 3A). However, Avastin treatment significantly suppressed VEGF density almost back to the control level. The density of hyperoxia-induced VEGF was reduced by the applied resveratrol in dose-dependent manners (Figure 3A). Resveratrol treatment via intravitreal injection and eye drops, both at high doses, decreased the density of VEGF by ~2-fold and ~1.5-fold, respectively; the applications of resveratrol, both at middle doses, weakened VEGF density down to ~3.2-fold (intravitreal injection) and ~4.2-fold (eye drops) (Figure 3A). In both intravitreal injection and eye drops, resveratrol showed similar inhibitory effects on VEGF density. All resveratrol-treated groups displayed significant suppressive effects in terms of hyperoxia-induced VEGF density (Figure 3A).

The density of VEGFR2 in retinal tissue was determined. On the basis of comparisons with the control group, the density of VEGFR2 expression in model group with no drug treatment was significantly increased by ~7-fold after hyperoxia (Figure 3B). Similarly, Avastin exerted suppressive effects on the hyperoxia-triggered VEGFR2 density from ~7-fold to ~2-fold, serving as a positive control. The application of resveratrol significantly reduced the density of VEGFR2 in a dose-dependent manner, and the maximal reduction was ∼3.5-fold that of the control via intravitreal injection at a high dose. Meanwhile, resveratrol application by intravitreal injection at low and middle doses markedly decreased the VEGFR2 density down to ~4-fold and ~3.5-fold, respectively (Figure 3B). In addition, the density of VEGFR2 was decreased by resveratrol treatment through eye drops; the VEGFR2 densities at low, middle and high doses were reduced to ~6.5-, ~6- and ~- fold, respectively (Figure 3B).

**Figure 4:**
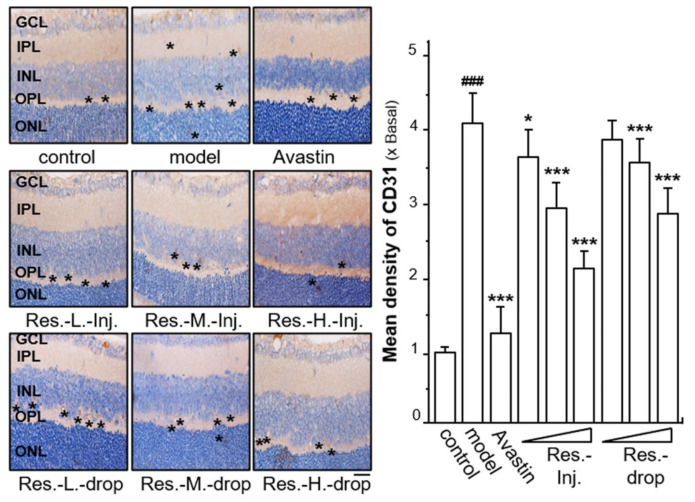
**Resveratrol suppresses hyperoxia-induced expressions of CD31 in retinal ROP model**. Retinal tissues were immuno-stained with the CD31 antibody and stained by DAB dye. The positive area is brown or yellowish brown (left). * shows the expressed part of the target protein. Image-Pro Plus version 6.0 software was used for the quantification of CD31 density (right). Results are expressed as the fold of change to control (x basal), where the control group (no drug) was set as 1, Mean ± SEM, where *n* = 6; ### *p* < 0.001 vs. the control group; * *p* < 0.05; *** *p* < 0.001 vs. the model group. Bar = 25 μm.

Immunohistochemistry for CD31 in retina was selected for determination, as CD31 is commonly regarded as a key endothelial cell marker. Compared with the control group, the hyperoxia-triggered group without drug treatment displayed a high vascular density of CD31 expression, increased by ~4-fold; however, the Avastin-treated group showed a significantly lower density of CD31, which decreased to almost the control level (Figure 4). Resveratrol exerted inhibitory effects on CD31 density in a dose-dependent manner, and with the same dose, the density of CD31 when treated with resveratrol via intravitreal injection was lower than in groups treated with resveratrol via eye drops (Figure 4). The treatment of resveratrol via intravitreal injection decreased the hyperoxia-induced CD31 density down to ~3.5-fold, ~3-fold and ~2-fold at low, middle and high doses, respectively. Similarly, the densities of CD31 expression in the groups treated with resveratrol by eye drops were reduced to ~4-fold, ~3.5-fold and ~3.0-fold at low, middle and high doses, respectively (Figure 4). However, low-dose resveratrol given via eye drops did not significantly suppress CD31 expression.

### 2.4. Resveratrol Relieves Oxidative Stress

**Figure 5:**
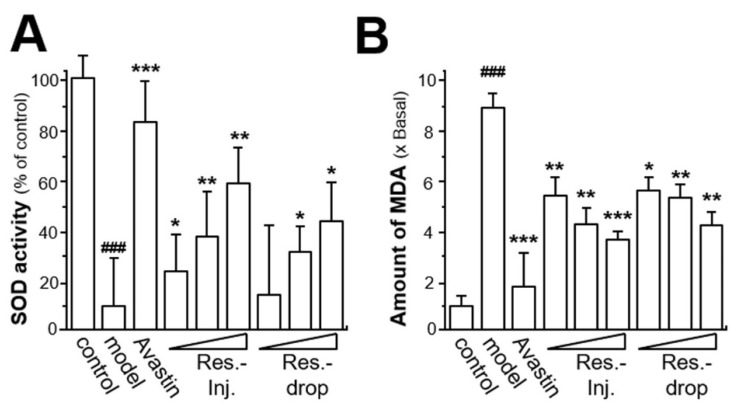
**Resveratrol releases oxidative stress by enhancing SOD activity and decreasing MDA activity.** (**A**) The quantification of SOD activity and (**B**) the quantification of MDA activity in the collected blood. The data are expressed as the percentage of control or the fold of change of control (x basal), in mean ± SEM, where *n* = 6; ### *p* < 0.001 vs. the control group; * *p* < 0.05; ** *p* < 0.01; *** *p* < 0.001 vs. the model group.

Oxidative stress has been demonstrated to be closely related with multiorgan failure. In the retinal area, oxidative stress is caused by the imbalance between production of pro-oxidants and detoxication of the harmful effects exerted by anti-oxidants. Nitric oxide, free radicals, hydrogen peroxide (H_2_O_2_) and superoxide anion contribute to this process [34]. SOD is not only a scavenging enzyme of superoxide anion, but also is the main producing enzyme of H_2_O_2_. SOD plays important roles in the oxidative stress system. Thus, the effects of resveratrol on the antioxidant system were determined by measuring SOD activity. In comparison with the control group, the hyperoxia decreased SOD activity by ~90% in the model group (Figure 5A). Compared with the model group, the treatment with a high dose of resveratrol, either by intravitreal injection or eye drops, could obviously relieve the activity of SOD in a dose-dependent manner, with maximal effects of upregulating SOD activity to ~40 to ~60% of the control (Figure 5A). At low and middle doses, resveratrol treatment by intravitreal injection significantly relieved the SOD activity to ~20% and ~35%, respectively. Application of resveratrol via eye drops, at low and middle doses, exerted potentiating effects on SOD activity by ~5% and ~30%, respectively (Figure 5A). As a positive control, compared with model group, Avastin application significantly relieved SOD activity to ~80% that of the control.

MDA is one of the final products of lipid peroxidation with high toxicity. MDA is a sensitive marker of oxidative stress, and is easily detectable in the retina. MDA may act as a potential mutagenic factor as it interacts with DNA and proteins [34]. Compared with control group, the hyperoxia treatment induced MDA activity up to ~9-fold in the model group, while Avastin treatment, serving as a positive control, significantly decreased MDA activity to ~2-fold that of the control (Figure 5B). The groups that were resveratrol-treated by intravitreal injection and eye drops displayed obviously reduced MDA activity to ~6-fold after a high dose (Figure 5B). At a middle dose, resveratrol treatment via intravitreal injection and eye drops significantly reduced MDA activity by ~4-fold and ~4.5-fold, respectively. Resveratrol via intravitreal injection at a low dose demonstrated similar inhibitory effects on MDA activity to that via eye drops, with a reduction of ~3.5-fold.

## 3. Discussion

Angiogenesis is controlled precisely, as abnormal angiogenesis contributes a lot to disease aggravation. VEGF is the key stimulating factor to induce angiogenesis by promoting growth of vascular endothelial cells [35], which promotes proliferation and migration of endothelial cells, developing complicated vascular networks. In the eye, VEGF contributes significantly to the progression of age-related macular degeneration (AMD), ROP, proliferative diabetic retinopathy and other neovascular disorders. In developing eye diseases, e.g., AMD and ROP, the level of VEGF is elevated, which suggests that inhibition of the VEGF-mediated angiogenesis could be a possible therapeutic approach for these eye diseases [36]. Indeed, anti-VEGF agents have been employed to suppress this VEGF-dependent vessel proliferation [37]. Targeting VEGF and its related signalling may be of great benefit in angiogenic modulation, alleviating disease symptoms [38]. According to the anti-VEGF agent, bevacizumab is a neutralizing antibody targeting VEGF protein; this antibody has been approved by Food and Drug Administration (FDA) for its application in metastatic colorectal cancer treatment, as well as in angiogenesis of various cancers [12,39,40]. With extensive clinical trials, the suppressive effects of bevacizumab on angiogenesis and vascular leakage have been proved to be effective in treating ocular diseases by intravitreal injection, such as AMD, ROP and diabetic retinopathy [41,42]. In addition, aptamers [43], peptides [44] and soluble decoy receptors [45] have been used clinically as anti-VEGF agents. Though these peptide-based drugs possess many advantages, such as low toxicity and high specificity, limited routes of administration and high costs have hindered their wide usage. Thus, the agents targeting VEGF with easy administration, e.g., resveratrol, with low-cost manufacturing, long-term administration and easy topical instillation, are of great benefit and should be developed.

Angiogenesis plays a major role in eye disease development: the morphological and functional changes in blood vessels are inducers of these angiogenic eye diseases. This also is a key pathological change in patients suffering from AMD, diabetic retinopathy and ROP [46]. VEGF is essential for the development of neural tissues and organs. During ROP treatment in preterm infants, organogenesis is still undergoing. In the eye, VEGF is essential for the development of a normal neural retina, as VEGF is highly expressed in these ocular diseases [47]. ROP is a major ocular complication and develops via hypoxia-triggered excessive VEGF expression and limited retinal oxygenation, thus leading to abnormal vaso-proliferation [18]. The pathologies of ROP development are not fully known; drugs for preventive and therapeutic treatments of ROP have limited application so far [23]. However, anti-VEGF therapy for the treatment of various kinds of ocular diseases, including ROP, has been shown to have positive results [18,23]. Thus, the inhibitors targeting VEGF may be of high therapeutic benefit.

Traditional Chinese medicine (TCM) is a great source for medicine development for the treatment of various diseases, and additionally, the application of botanical dietary supplements is very attractive and promising. However, the identification of active compounds from herbal mixtures hinders the internationalization of TCM, as well as their possible therapeutic purposes. In our previous reports, we have identified a number of phytochemicals with anti-angiogenic effects, e.g., polydatin [29], resveratrol [30], and kaempferol [48]. These compounds exerted functionality via binding with VEGF at the heparin binding site, or at the receptor activation site of the molecule, to block the binding of VEGF to its receptor. In addition, the synergistic effects of a combination of ginkgetin and resveratrol in angiogenic modulation were also proposed [49]. In view of VEGF playing role in angiogenesis-related diseases, e.g., macular degeneration, psoriasis and tumour proliferation, the aforementioned natural compounds that attenuated VEGF-induced angiogenesis could be promising regarding the development of relevant drugs for angiogenesis-related disease treatment. Inspired by the previous studies, we proceeded our exploration in testing resveratrol for inhibition of retinal angiogenesis during the development of ROP in mice.

Resveratrol is a rather safe phytochemical, commonly found in many natural and edible plants, e.g., grapes, pines and peanuts. The application of resveratrol for the prevention and treatment of various metabolic disorders has been proposed [50], and this compound has been demonstrated to have inhibitory activities on VEGF-mediated angiogenesis [30]. Here, we hypothesized that resveratrol exerted suppressive effects on retinal angiogenesis and could be used to treat eye diseases. In the early progression of ROP, the breakdown of the blood–retinal barrier appears to be caused by developing macular oedema and increasing vascular permeability, and therefore visual impairment is induced [51]. Here, resveratrol was shown to exert inhibitory effects in retinal angiogenesis either by intravitreal injection or topical instillation. This notion is fully supported by (i) restoration of vascular distortion; and (ii) enhancement of vascular permeability. In addition, resveratrol further significantly inhibited pathologic retinal angiogenesis in an ROP model by either intravitreal injection or topical instillation, which decreased the extent of vascular hyperplasia and the formation of abnormal vascular tufts. Moreover, the application of resveratrol obviously attenuated the expressions of VEGF, VEGFR2 and CD31, suggesting the suppressive effects of resveratrol in VEGF signalling and retinal angiogenesis. In general, resveratrol given by intravitreal injection demonstrated better effects in inhibiting retinal angiogenesis than when given by topical instillation, which may be attributed to the fact that resveratrol given by intravitreal injection can more easily be circulated in the blood. This notion however needs further investigation.

ROS is formed in mitochondria, playing a key role in retinal vaso-obliteration of ROP [52]. SOD is of vital importance in the antioxidant defence of photoreceptors, and reduces oxidative damage in the retina by catalysing ROS, including H_2_O_2_, peroxynitrite and superoxide anion [53]. MDA appears to be a promising target for diagnosis and treatment of ROP, because it has been shown to be involved mainly in the cascade of oxidative stress damage. Indeed, oxidative stress has been proposed to be a major molecular mechanism contributing to ROP pathogenesis [34]. Thus, SOD and MDA are drug targets in the prevention of retinal damage and irreversible vision disorders. The current results showed that resveratrol given by intravitreal injection and topical instillation could significantly relieve the damage caused by oxygen radicals through upregulating the level of SOD and downregulating the level of MDA. These could be parameters considered for ROP treatment.

## 4. Materials and Methods

### 4.1. Reagents and Animals

Resveratrol was purchased from Chengdu Institute of Biology (Chengdu, China), and the purity was >98%, detected by HPLC with diode-array detection. Avastin (bevacizumab), a recombinant humanized monoclonal antibody against VEGF blocking angiogenesis, was purchased from Genentech (San Francisco, CA, USA). The following antibodies (VEGF; VEGFR2 (Tyr1175) (19A10): VEGFR2 and CD31 (cluster of differentiation 31)), were purchased from Cell Signaling Technology (Danvers, MA, USA). Hematoxylin, eosin and Evans blue were bought from Sigma-Aldrich (St. Louis, MO, USA). Superoxide dismutase (SOD) activity and lipid peroxidation assay kits were purchased from Abcam (Cambridge, UK). Other reagents were of analytical purity. The mouse protocol was approved by Hangzhou Hibio Experimental Animal Ethics Committee (Permit Number: HB1808021) under the guidelines of the “Principles of Laboratory Animal Care” (NIH publication No. 80-23, revised 1996) and Institutional Animal Care and Use Committees protocol (HBFM3.68-2015) (Figure S1).

### 4.2. Oxygen-Induced Retinopathy

ROP is characterized by abnormal retinal neovascularization, which is similar to the vascular proliferation in wet age-related macular degeneration (AMD). An oxygen-induced retinopathy mouse model was developed, referring to a previous report [33]. For ROP model establishment, C57BL/6 suckling mice (7 days old) were exposed to a hypoxic/hyperoxic chamber for 5 days. The oxygen partial pressure was at normal pressure. The oxygen concentration was set at (75 ± 2)% in an oxygen chamber. During this period, an oxygen meter was used to monitor the oxygen concentration in the container 4–6 times a day. Food, water and cushion material were changed regularly every day. After 5 days, the suckling mice were returned to a normal air environment for 5 days. The mice were randomly divided into 9 groups: control group, model group, Avastin group (20 mg/kg), resveratrol low-dosage group (intravitreal injection, 5 mg/kg), resveratrol middle-dosage group (intravitreal injection, 25 mg/kg), resveratrol high-dose group (intravitreal injection, 50 mg/kg), resveratrol low-dosage group (eye drop, 5 mg/kg), resveratrol middle-dosage group (eye drop, 25 mg/kg/day), and resveratrol high-dose group (eye drop, 50 mg/kg/day). Here, the ‘model group’ is referring to the oxygen-induced retinopathy mouse model without any drug treatment. Each group consisted of ten suckling mice. Avastin was dissolved in saline and administered through intravitreal injection twice a day. Resveratrol was dissolved in a PEG-400 and sunflower oil mixture. Resveratrol was administrated by intravitreal injection or topical instillation (eye drops) at the indicated concentrations after being diluted by normal saline. Drug treatment lasted for 5 days, and resveratrol was administered twice a day. The body weight of mice was measured every day throughout the period of treatment. All animals were fed a normal diet with free access to water in specific pathogen-free animal facility. Samples were taken from 17 days after birth for follow-up tests.

### 4.3. Collection of Eyes and Serum

Both eyes from mice in each group were enucleated and placed on ice. Ice-cold phosphate-buffered saline (PBS) was used to rinse the eyes. The pH value of PBS was set to 7.4. The vitreous fluid was processed [54]. Vitreous fluid was first aspirated by using a 0.5 mL insulin syringe and then placed on ice in sterile Eppendorf tubes. Vitreous fluid was collected for each group without contamination by blood. Retinas were firstly excised and placed in sterile polypropylene tubes on ice. Snap freezing was finally performed to freeze retinas. Each sample containing vitreous fluid or retina was collected. Retinas were pooled to obtain enough samples for measurement of retinal vascular permeability, immunohistochemistry and antioxidant activity assays. Retinas were assessed using Evans blue, diaminobenzidine (DAB) and hematoxylin and eosin (H&E) staining. After decapitation, blood samples from each mouse were separately collected into sterile Eppendorf tubes. Blood samples were placed on ice and allowed to clot for half an hour before processing. For serum collection, the tubes were centrifuged at 2000× *g* for 20 min. Serum and vitreous samples were kept in −20 °C, and retinal samples were stored in −80 °C until use.

### 4.4. Measurement of Retinal Vascular Permeability

Evans blue dye is commonly used as nonradioactive intravascular tracer. Evans blue dye rapidly binds to plasma albumin after intravenous injection, and thus remains within the vasculature. Evans blue may leak out into the surrounding tissues if plasma extravasation occurs. Therefore, the quantity of Evans blue can be used as a marker for plasma extravasation [55]. Evans blue extravasation technique was applied to assess retinal vascular permeability. Mice were anesthetized using ketamine (50 mg/kg) and xylazine hydrochloride (100 mg/kg). Then, a PE-50 polyethylene catheter (Beckton Dickinson, Sparks, MD, USA) was inserted into the right femoral vein. Evans blue (45 mg/mL in saline) was injected intravenously over 10 s. The mice turned visibly blue immediately after infusion of Evans blue, demonstrating the success uptake and distribution of the dye. The circulation of Evans blue lasted for two hours. To wash out intravascular dye, saline was infused through the left ventricle and the retina was dissected out after enucleation. To compare retinal vascular permeability between stained and non-stained areas, Evans blue was extracted by incubating each retina in 1 mL formamide for eighteen hours at 70–75 °C. Pictures were taken under an Olympus BX43 microscope (Olympus, Center Valley, PA, USA) with excitation wavelength set at 620 nm and emission wavelength set at 680 nm. Images of the retinas were presented at 20× magnification. To measure the leakage area, images were imported into Image-Pro Plus 6.0 software for grey value analysis. After elimination of the strong light area in the blood vessel, the area with grey values between 230 and 255 was determined as the leakage area. The visual field and seepage areas were derived. The ratio of visual field area and seepage area represented the percentage of leakage area [56].

### 4.5. Retinal Hematoxylin and Eosin Staining

Retinal sections were immersed in ethanol solutions for dehydration. The concentrations of ethanol used here were 80, 95 and 100%. Next, sections were embedded in paraffin for 5 h and solidified, then paraffin-embedded tissues were then kept in −20 °C until frozen. Then, the frozen paraformaldehyde/paraffin-embedded samples were sectioned into 4 μm-thick slices, and the sliced tissues were mounted on microscope slides. During sectioning, the eyes were oriented with the cornea in parallel to the optic nerve, and the optic nerve was visible in the sections. Following 5 min of PBS washing three times, slides were de-paraffinized using xylene and hydrated using successive alcohol (100, 90, and 70%) and distilled water. The slides were washed 3 times by PBS. Three × 5 min PBST washes were performed on the sections. The slides were incubated with haematoxylin for 15 min at room temperature and washed using distilled water for 1 min. Slides were immersed in 1% hydrochloric acid–alcohol complex solution until sections turned blue. The slides were incubated with eosin for 5 min and distilled water was used to wash off the floating colour. After dying, hydration was performed by sequential immersion of slides in xylene and successive alcohol solutions (80, 95, and 100%). Finally, slides were sealed by using neutral gum. Images were taken under a light BX43 microscope (Olympus). Data were generated using Zen and Image J software to analyse images of vascular hyperplasia regions in inner plexiform layer (IPL), inner nuclear layer (INL) and outer plexiform layer (OPL). These data supported the comparison between the central and peripheral retina.

### 4.6. Immunohistochemistry

Mouse retina tissue was fixed in 4% paraformaldehyde solution for 24 h. After fixation, tissues were taken out from the fixative and tripped into an appropriate shape. Next, tissues were successively dehydrated using 80% ethanol, 90% ethanol, 95% ethanol and 100% ethanol and embedded in paraffin for 5 h. After they solidified, the paraffin-embedded tissues were kept at −20 °C. The paraformaldehyde/paraffin-embedded samples were sectioned into 4 μm-thick slices. Sliced tissues were mounted on microscope slides. Slides were de-paraffinized using xylene and hydrated by graded alcohol. Slides were washed 3 times with PBS. To block and inactivate endogenous peroxidase, slides were processed by applying 3% H_2_O_2_, and blocked for half an hour at 37 °C. Antigen retrieval was undertaken by heating for 10 min in 0.01 M sodium citrate buffer. The pH value of sodium citrate buffer was set at 6.0. Tissue sections were incubated with primary antibody at a 1:1000 dilution at 4 °C overnight. After washing in 1× PBS three times, the samples were incubated with anti-rabbit secondary antibody at a 1:500 dilution for 2 h at room temperature. Diaminobezidin (DAB) dye reaction followed, and the reaction progress was observed under a microscope. After washing in deionized water, hematoxylin counterstaining was performed. After drying and sealing, the sections were observed under a light BX43 microscope (Olympus). Images were quantified by Image-Pro Plus version 6.0 software (Media Cybernetics, Rockville, MD, USA).

### 4.7. Antioxidant Activity

Blood from mice was collected by using EDTA. The blood mixture was centrifuged at 1000× *g* for 10 min at 4 °C. After centrifuging, the serum layer was transferred to a new tube without disturbing the buffer layer. The serum was stored at −80 °C until further analysis. SOD and malondialdehyde (MDA) activities in the homogenates were determined using commercially available SOD and MDA assay kits, following the manufacturer’s protocol (Abcam, Cambridge, UK). The activity of SOD was measured by using 2,4 iodophenyl 3,4 nitrophenol 5 phenyltetrazolium chloride, as SOD exerts inhibitory effects on INT/formazan dye with wavelength set at 505 nm [57]. Serum MDA activity was determined by applying the colorimetric method, as previously described [58]. As one of the aldehyde products of lipid peroxidation, MDA reacted with thiobarbituric acid (TBA), and the MDA-TBA adduct was produced. The absorbance of the MDA-TBA adduct was determined by spectrophotometry with a wavelength of 532 nm.

### 4.8. Other Assays

Protein concentration was measured by the Bradford method with a kit from Bio-Rad (Hercules, CA, USA).

### 4.9. Statistical Analysis

One-way analysis of variance (ANOVA) was performed for statistical analysis to determine differences among groups. A Bonferroni multiple comparisons test was performed by applying the SPSS 16.0 software. Significance was set at *p* < 0.05, and data were reported as the mean ± standard error of the mean (SEM).

## 5. Conclusions

This study explores the potential application of resveratrol in treating angiogenic-related eye diseases, e.g., ROP and AMD. The clinical usage of resveratrol has major advantages regarding cost, safety and convenient application via eye drops. Besides the blocking of VEGF function, resveratrol can prevent oxygen radical damage. Moreover, resveratrol-containing eye drops can even be used without the presence of eye problems, and therefore could be developed as a preventive measure.

## Data Availability

Not applicable.

## Supplementary Materials

The following supporting information can be downloaded at: https://www.mdpi.com/article/10.3390/ijms23126455/s1.

## References

[1] Ferrara N., Kerbelb R.S. (2005). Angiogenesis as a therapeutic target. Nature.

[2] Hanahan D., Folkman J. (1996). Patterns and emerging mechanisms of the angiogenic switch during tumorigenesis. Cell.

[3] Aiello L.P. (2005). Angiogenic pathways in diabetic retinopathy. N. Engl. J. Med..

[4] Gariano R.F., Gardner T.W. (2005). Retinal angiogenesis in development and disease. Nature.

[5] Cursiefen C., Chen L., Saint-Geniez M., Hamrah P., Jin Y., Rashid S., Pytowski B., Persaud K., Wu Y., Streilein J.W. (2006). Nonvascular VEGF receptor 3 expression by corneal epithelium maintains avascularity and vision. Proc. Natl. Acad. Sci. USA.

[6] Cursiefen C., Rummelt C., Junemann A., Vorwerk C., Neuhuber W., Kruse F.E., Schroedl F. (2006). Absence of blood and lymphatic vessels in the developing human cornea. Cornea.

[7] Bock F., Onderka J., Dietrich T., Bachmann B., Kruse F.E., Paschke M., Zahn G., Cursiefen C. (2007). Bevacizumab as a potent inhibitor of inflammatory corneal angiogenesis and lymphangiogenesis. Investig. Ophthalmol. Vis. Sci..

[8] Samolov B., Steen B., Seregard S., van der Ploeg I., Montan P., Kvanta A. (2005). Delayed inflammation-associated corneal neovascularization in MMP-2-deficient mice. Exp. Eye Res..

[9] Cursiefen C., Maruyama K., Jackson D.G., Streilein J.W., Kruse F.E. (2006). Time course of angiogenesis and lymphangiogenesis after brief corneal inflammation. Cornea.

[10] Chang J.H., Gabison E.E., Kato T., Azar D.T. (2001). Corneal neovascularization. Curr. Opin. Ophthalmol..

[11] Leung D.W., Cachianes G., Kuang W.J., Goeddel D.V., Ferrara N. (1989). Vascular endothelial growth factor is a secreted angiogenic mitogen. Science.

[12] Ferrara N. (2004). Vascular endothelial growth factor: Basic science and clinical progress. Endocr. Rev..

[13] Penn J.S., Madan A., Caldwell R.B., Bartoli M., Caldwell R.W., Hartnett M.E. (2008). Vascular endothelial growth factor in eye disease. Prog. Retin. Eye Res..

[14] Phillips G.D., Stone A.M., Jones B.D., Schultz J.C., Whitehead R.A., Knighton D.R. (1994). Vascular endothelial growth factor (rhVEGF165) stimulates direct angiogenesis in the rabbit cornea. In Vivo.

[15] Philipp W., Speicher L., Humpel C. (2000). Expression of vascular endothelial growth factor and its receptors in inflamed and vascularized human corneas. Investig. Ophthalmol. Vis. Sci..

[16] Iwasaki J., Nihira S. (2009). Anti-angiogenic therapy against gastrointestinal tract cancers. Jpn. J. Clin. Oncol..

[17] Manzano R.P., Peyman G.A., Khan P., Carvounis P.E., Kivilcim M., Ren M., Lake J.C., Chevez-Barrios P. (2007). Inhibition of experimental corneal neovascularisation by bevacizumab (Avastin). Br. J. Ophthalmol..

[18] Hellstrom A., Smith L.E.H., Dammann O. (2013). Retinopathy of prematurity. Lancet.

[19] Raju T.N., Langenberg P., Bhutani V., Quinn G.E. (1997). Vitamin E prophylaxis to reduce retinopathy of prematurity: A reappraisal of published trials. J. Pediatr..

[20] Connor K.M., SanGiovanni J.P., Lofqvist C., Aderman C.M., Chen J., Higuchi A., Hong S., Pravda E.A., Majchrzak S., Carper D. (2007). Increased dietary intake of omega-3-polyunsaturated fatty acids reduces pathological retinal angiogenesis. Nat. Med..

[21] Lee J., Kim K.E., Choi D.K., Jang J.Y., Jung J.J., Kiyonari H., Shioi G., Chang W., Suda T., Mochizuki N. (2013). Angiopoietin-1 guides directional angiogenesis through integrin alphavbeta5 signaling for recovery of ischemic retinopathy. Sci. Transl. Med..

[22] Chen J., Connor K.M., Aderman C.M., Smith L.E. (2008). Erythropoietin deficiency decreases vascular stability in mice. J. Clin. Investig..

[23] Sapieha P., Joyal J.S., Rivera J.C., Kermorvant-Duchemin E., Sennlaub F., Hardy P., Lachapelle P., Chemtob S. (2010). Retinopathy of prematurity: Understanding ischemic retinal vasculopathies at an extreme of life. J. Clin. Investig..

[24] Micieli J.A., Surkont M., Smith A.F. (2009). A systematic analysis of the off-label use of bevacizumab for severe retinopathy of prematurity. Am. J. Ophthalmol..

[25] Parikh R., Ross J.S., Sangaralingham L.R., Adelman R.A., Shah N.D., Barkmeier A.J. (2017). Trends of anti-vascular endothelial growth factor use in ophthalmology among privately insured and medicare advantage patients. Ophthalmology.

[26] Smith A.G., Kaiser P.K. (2014). Emerging treatments for wet age-related macular degeneration. Expert Opin. Emerg. Drugs.

[27] Gunay M., Sukgen E.A., Celik G., Kocluk Y. (2016). Comparison of Bevacizumab, Ranibizumab, and Laser Photocoagulation in the Treatment of Retinopathy of Prematurity in Turkey. Curr. Eye Res..

[28] Nicoara S.D., Cristian C., Irimescu I., Stefanut A.C., Zaharie G. (2014). Diode laser photocoagulation for retinopathy of prematurity: Outcomes after 7 years of treatment. J. Pediatr. Ophthalmol. Strabismus.

[29] Hu W.H., Wang H.Y., Kong X.P., Xiong Q.P., Poon K.M., Xu L., Duan R., Chan K.L., Dong T.X., Tsim W.K. (2019). Polydatin suppresses VEGF-induced angiogenesis through binding with VEGF and inhibiting its receptor signalling. FASEB J..

[30] Hu W.H., Duan R., Xia Y.T., Xiong Q.P., Wang H.Y., Chan G.L., Liu S.Y., Dong T.X., Qin Q.W., Tsim W.K. (2019). The binding of resveratrol to vascular endothelial growth factor (VEGF) suppresses angiogenesis by inhibiting the receptor signalling. J. Agric. Food Chem..

[31] Hu W.H., Chan G.K., Lou J.S., Wu Q.Y., Wang H.Y., Duan R., Cheng M.Y., Dong T.X., Tsim W.K. (2018). The extract of Polygoni Cuspidati Rhizoma et Radix suppresses the vascular endothelial growth factor-induced angiogenesis. Phytomedicine.

[32] Stahl A., Lepore D., Fielder A., Fleck B., Reynolds J.D., Chiang M.F., Li J., Liew M., Maier R., Zhu Q. (2019). Ranibizumab versus laser therapy for the treatment of very low birthweight infants with retinopathy of prematurity (RAINBOW): An open-label randomised controlled trial. Randomized Control. Trial.

[33] Villacampa P., Menger K.E., Abelleira L., Ribeiro J., Duran Y., Smith A.J., Ali R.R., Luhmann U.F. (2017). Bainbridge JWB. Accelerated oxygen-induced retinopathy is a reliable model of ischemia-induced retinal neovascularization. PLoS ONE.

[34] Banjac L., Banjac G., Kotur-Stevuljevi J., Spasojevi-Kalimanovska V., Gojkovic T., Bogavac-Stanojevi’c N., Jelic-Ivanovi’c Z., Banjac G. (2018). Pro-oxidants and antioxidants in retinopathy of prematurity. Acta Clin. Croat..

[35] Bikfalvi A., Sauzeau C., Moukadiri H., Maclouf J., Busso N., Bryckaert M., Plouet J., Tobelem G. (1991). Interaction of vasculotropin/vascular endothelial cell growth factor with human umbilical vein endothelial cells: Binding, internalization, degradation, and biological effects. J. Cell Physiol..

[36] Kliffen M., Sharma H.S., Mooy H.S., Kerkvliet C.M., de Jong P.T.V.M. (1997). Increased expression of angiogenic growth factors in agerelated maculopathy. Br. J. Ophthalmol..

[37] Kwak N., Okamoto N., Wood J.M., Campochiaro P.A. (2000). VEGF is major stimulator in model of choroidal neovascularization. Investig. Ophthalmol. Vis. Sci..

[38] Ellis L.M., Hicklin D.J. (2008). VEGF-targeted therapy: Mechanisms of anti-tumour activity. Nat. Rev. Cancer.

[39] Hurwitz H., Fehrenbacher L., Novotny W., Cartwright T., Hainsworth J., Heim W., Berlin J., Baron A., Griffing S., Holmgren E. (2004). Bevacizumab plus irinotecan, fluorouracil, and leucovorin for metastatic colorectal cancer. N. Engl. J. Med..

[40] Sennino B., McDonald D.M. (2012). Controlling escape from angiogenesis inhibitors. Nat. Rev. Cancer.

[41] Arevalo J.F., Wu L., Sanchez J.G., Maia M., Saravia M.J., Fernandez C.F., Evans T. (2009). Intravitreal bevacizumab (Avastin) for proliferative diabetic retinopathy: 6-months follow-up. Eye.

[42] Avery R.L., Pieramici D.J., Rabena M.D., Castellarin A.A., Nasir M.A., Giust M.J. (2006). Intravitreal bevacizumab (Avastin) for neovascular age-related macular degeneration. Ophthalmology.

[43] Ng E.W., Shima D.T., Calias P., Cunningham E.T.J., Guyer D.R., Adamis A.P. (2006). Pegaptanib, a targeted anti-VEGF aptamer for ocular vascular disease. Nat. Rev. Drug Dis..

[44] Bikfalvi A. (2004). Recent developments in the inhibition of angiogenesis: Examples from studies on platelet factor-4 and the VEGF/VEGFR system. Biochem. Pharmacol..

[45] Holash J., Davis S., Papadopoulos N., Croll S.D., Ho L., Russell M., Boland P., Leidich R., Hylton D., Burova E. (2002). VEGFTrap: A VEGF blocker with potent antitumor effects. Proc. Natl. Acad. Sci. USA.

[46] Wells J.A., Murthy R., Chibber R., Nunn A., Molinatti P.A., Kohner E.M., Gregor Z.J. (1996). Levels of vascular endothelial growth factor are elevated in the vitreous of patients with subretinal neovascularisation. Br. J. Ophthalmol..

[47] Sato T., Kusaka S., Shimojo H., Fujikado T. (2009). Vitreous levels of erythropoietin and vascular endothelial growth factor in eyes with retinopathy of prematurity. Ophthalmology.

[48] Hu W.H., Wang H.Y., Dai K., Zheng Z.Y., Xiong Q.P., Dong T.X., Duan R., Chan G.K., Bi W.C., Tsim W.K. (2020). Kaempferol, a major flavonoid in Ginkgo Folium, binds to vascular endothelial growth factor and potentiates angiogenic functions in cultured endothelial cells. Front. Pharmacol..

[49] Hu W.H., Chan G.K., Duan R., Wang H.Y., Kong X.P., Dong T.X., Tsim W.K. (2019). Synergy of ginkgetin and resveratrol in suppressing VEGF-induced angiogenesis: A therapy in treating colorectal cancer. Cancers.

[50] Kershaw J., Kim K.H. (2017). The therapeutic potential of piceatannol, a natural stilbene, in metabolic diseases: A review. J. Med. Food.

[51] Joussen A.M., Smyth N., Niessen C. (2007). Pathophysiology of diabetic macular edema. Dev. Ophthalmol..

[52] Hardy P., Beauchamp M., Sennlaub F., Gobeil F.J., Mwaikambo B., Lachapelle P., Chemtob S. (2005). Inflammatory lipid mediators in ischemic retinopathy. Pharmacol. Rep..

[53] Rao N.A., Thaete L.G., Delmage J.M., Sevanian A. (1985). Superoxide dismutase in ocular structures. Invest. Ophthalmol. Vis. Sci..

[54] Brock R.S., Gebrekristos B.H., Kuniyoshi K.M., Modanlou H.D., Falcao M.C., Beharry K.D. (2011). Biomolecular effects of JB1 (an IGF-I peptide analog) in a rat model of oxygen-induced retinopathy. Pediatr. Res..

[55] Abdouh M., Talbot S., Couture R., Hassessian H.M. (2008). Retinal plasma extravasation in streptozotocin-diabetic rats mediated by kinin B_1_ and B_2_ receptors. Br. J. Pharmacol..

[56] Higgins R.D., Yu K., Sanders R.J., Nandgaonkar B.N., Rotschild T., Rifkin D.B. (1999). Diltiazem reduces retinal neovascularization in a mouse model of oxygen induced retinopathy. Curr. Eye Res..

[57] Beauchamp C., Fridovich I. (1971). Superoxide dismutase: Improved assays and an assay applicable to acrylamide gels. Anal. Biochem..

[58] Placer Z.A., Lind L., Cushmann M., Johnson B.C. (1966). Estimation of product of lipid peroxidation (MDA) in biological systems. Anal. Biochem..

