# Supplementary material for: Resveratrol, an Inhibitor Binding to VEGF, Restores the Pathology of Abnormal Angiogenesis in Retinopathy of Prematurity (ROP) in Mice: Application by Intravitreal and Topical Instillation
Source: Int J Mol Sci. 2022 Jun 9;23(12):6455. doi: 10.3390/ijms23126455 (PMC9223486; doi:10.3390/ijms23126455)
Supplement: Supplementary file 1 [file ijms-23-06455-s001.zip › Supplementary figure-Hu et al. 2022.pdf]

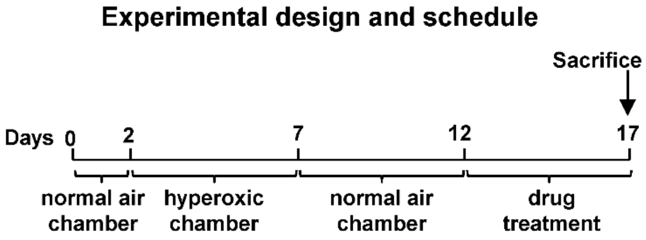

Supplementary Figure S1. The schedule of animal experiment.

C57BL/6 suckling mice (2 days old) were used to establish ROP model as exposed to hypoxic/hyperoxic chamber for 5 days. The oxygen partial pressure was normal pressure. The oxygen concentration was set at (75 ± 2) %. After 5 days, all the suckling mice were returned to normal air environment for another 5 days. Thereafter, mice were randomly divided into 9 groups: the control group, model group, Avastin group (20 mg/kg), resveratrol low-dosage group (intravitreal injection, 5 mg/kg), resveratrol middle-dosage group (intravitreal injection, 25 mg/kg), resveratrol high-dose group (intravitreal injection, 50 mg/kg), resveratrol low-dosage group (eye drop, 5 mg/kg), resveratrol middle-dosage group (eye drop, 25 mg/kg/day), resveratrol high-dose group (eye drop, 50 mg/kg/day). Each group consisted of ten suckling mice. Avastin was dissolved in saline and administered through intravitreal injection twice a day. Drug treatment lasted for 5 days, twice a day. In mice control group and mice model group, the mice were injected with the corresponding volume of saline. Mice were sacrificed from 17 days after birth for follow-up tests.
